# Therapeutical Notes

**Published:** 1899-03-11

**Authors:** 


### THERAPEUTICAL NOTES.

### The Treatment of Neuralgia.

The *Clinica Moderna* recommends the following :—

℞ Extract of Cannabis Indica, grains 7½.
Salicylic acid, grains 75.
M. Make into 10 powders.

M. et Signe. Two or three powders to be taken daily.
—N.Y. *Med. Jour.*

### Euchinine in Malaria.

St. George Gray, of St. Lucia, recommends euchinine instead of ordinary quinine, on account of its tastelessness. He finds it is more powerful as an antipyretic than quinine, and that 10—15 grains are as efficacious as 20 to 30 grains of sulphate of quinine. He never exceeds 15 grains once or twice a day, always commencing with a good purge, and in these doses it produces buzzing in the ears in many cases. The absence of the bitter taste is with many persons a great recommendation.—*Brit. Med. Journal.*

